# Early morpho-physiological response of oilseed rape under seed applied Sedaxane fungicide and *Rhizoctonia solani* pressure

**DOI:** 10.3389/fpls.2023.1130825

**Published:** 2023-02-22

**Authors:** Anna Panozzo, Giuseppe Barion, Selina Sterup Moore, Francesca Cobalchin, Alberto Di Stefano, Luca Sella, Teofilo Vamerali

**Affiliations:** ^1^ Department of Agronomy, Food, Natural Resources, Animals and the Environment, University of Padua, Padova, Italy; ^2^ Department of Land, Environment, Agriculture and Forestry, University of Padua, Padova, Italy

**Keywords:** biostimulant, hormone-like activity, photosynthesis, root growth, shoot phytotoxicity

## Abstract

The SDHI fungicide Sedaxane has shown to efficiently control *Rhizoctonia* spp. growth and to possess biostimulant properties in cereal crops. As a first, the present study investigated its effectiveness as a seed treatment of the dicot species oilseed rape (*Brassica napus* var. *oleifera*). For this, seeds were treated with different fungicides: *(i)* the conventionally used active ingredient Thiram, *(ii)* Sedaxane, or (*iii)* Sedaxane in combination with Fludioxonil and Metalaxyl-M, and later sown in soil inoculated with *Rhizoctonia solani*. The resulting shoot and root growth from the treated seeds were recorded in early growth stages and the presence of Rhizoctonia DNA in the basal stem tissue was quantified. Here we demonstrate that all the fungicide treatments were effective in greatly reducing the presence of Rhizoctonia DNA, with Thiram confirming to have high fungicidal effects. Following seed treatment, shoot and root growth at the 2-leaf stage was reduced regardless of inoculation, indicating that the fungicides became phytotoxic, with particular respect to Thiram. In seedlings grown in inoculated soil, significant biostimulation of the roots was observed at the 4-leaf stage of treatments containing both Sedaxane alone and in a mixture. Leaf area was stimulated in control soil not inoculated with *Rhizoctonia*, likely due to improved PSII efficiency, stomatal conductance, and CO_2_ assimilation rate. Young oilseed rape seedlings are thus highly sensitive to seed treatments with these fungicides, and in particular to Thiram. The retardation in growth is quickly overcome by the 4-leaf stage however. We confirm that Sedaxane indeed possesses root biostimulant properties in oilseed rape, which are enhanced in combination with other fungicides. Such biostimulating properties impose its greatest effects under conditions of biotic stress.

## 1 Introduction

Seed coating with fungicides is known to provide effective protection from soil and seed-borne pathogens during germination and early growth stages of field crops (Mathre et al., 2001). Modern fungicide formulations for seed dressing often constitute a mixture of active ingredients (a.i.) each with different modes of action, thereby contrasting a wide range of pathogens (Kitchen et al., 2016). The soil-borne *Rhizoctonia solani* Kühn fungus (teleomorph: *Thanatephorus cucumeris*) is one of the major pathogens of oilseed rape (Huber et al., 1992). It is responsible for significant yield losses caused by reduced seed germination and plant growth (Acharya et al., 1984; Gugel et al., 1987). Oilseed rape can be infected by two different anastomosis groups (AGs) of *R. solani*, namely AG2-1 and AG4, which are both associated to damping-off, root rot and yield reductions. AG2-1 is the most virulent, aggravating oil production losses of up to 30% (Tahvonen et al., 1984; Kataria and Verma, 1992; Khangura et al., 1999). *R. solani* mycelium, which originates from wintering sclerotia, grows towards the plant and penetrates the epidermal cells of the roots and stem base (Sherwood, 1970; Armentrout and Downer, 1987). The fungi can enter the plant either passively through wounds, lenticels and stomata (Nakayama, 1940; Murray, 1982), or actively, *via* enzymatic and mechanical pathways (Trail and Kolller, 1990).

In recent years, various active ingredients (a.i.) have been made commercially available to implement chemical control of *Rhizoctonia*. Metalaxyl-M is effective against both *Fusarium* spp. and *R. solani* in oilseed rape. It reduces damping-off during germination by inhibiting RNA polymerase activity *via* a systemic translocation pathway (Hwang et al., 2007). Seed application of the a.i. Fludioxonil is associated with a reduction of conidial germination in several fungi, such as snow mould (*Microdochium nivale*), *Botrytis cinerea* and *Penicillium expansum*. Fludioxonil inhibits the transport-associated phosphorylation of glucose and prevents glycerol synthesis (Leroux, 1996; Errampalli, 2004; Knauf-Beiter and Zeun, 2012). The combination of various fungicides has successfully been applied in various cereal crops, as for example the mixture Metalaxyl-M and Fludioxonil against root rot in buckwheat (Mondal, 2004). Nonetheless, some of these effective a.i. have already been withdrawn from the market in various countries (e.g., Thiram) or are in immediate risk of being revoked (e.g., Fludioxonil) due to their environmental impact and toxicity, therefore it is needed to search for new a.i.

Besides identifying the highly effective a.i. of fungicides, there has been a growing interest in exploring their plant biostimulant properties as supplementary effects when used in seed or leaf treatments. Fungal compounds conferring biostimulant effects relating to improved tolerance to abiotic stresses have already been discovered and characterised in various fungicides, including the ubiquinol oxidase inhibitors (Qol) strobilurins and azoles (Berdugo et al., 2012). The biostimulant effects of Sedaxane, a fungicide recently released on the market and classified as a pyrazole-carboxamide succinate dehydrogenase inhibitor (SDHI) have also been demonstrated (Zeun et al., 2013; Ajigboye et al., 2016). Sedaxane has shown to be able to efficiently control both seed- and soil-borne pathogenic fungi infection and growth, while also improving root growth, drought tolerance and shoot biomass in various cereal crops (Swart, 2011; Dal Cortivo et al., 2017). However, little is known on its concurrent effectiveness as a fungicide and biostimulant agent when used as a seed treatment in dicotyledonous species.

Given this background, the goals of this study were to (i) discriminate the antifungal efficacy against *R. solani* versus the biostimulant effect of Sedaxane in the dicotyledonous oilseed rape (*Brassica napus* var. *oleifera*), in comparison with the conventional fungicide Thiram (withdrawn since 2019 in Italy); (ii) assess whether the application of Sedaxane in combination with other active ingredients has greater efficacy as compared to Sedaxane alone in both controlling the pathogen and promoting plant growth; and (iii) highlight any physiological response, particularly relating to photosynthesis, to the tested fungicides in young oilseed rape plants. To achieve these goals, Sedaxane was applied as seed treatment on oilseed rape alone or in combination with Fludioxonil and Metalaxyl-M and compared to seeds treated with Thiram. The effects were measured in early growth stages of plants grown in rhizoboxes (2-leaf) and pots (4-leaf) containing soil inoculated with *R. solani* and compared to controls not inoculated. Root (depth, surface area and diameter, biomass) and shoot (leaf area and biomass, photosynthetic activity) parameters were assessed at plant harvest together with the amount of DNA of *R. solani* in the stem base tissues of oilseed rape through quantitative PCR (qPCR) analysis.

## 2 Materials and methods

### 2.1 Rhizobox and pot trials set-up

Plants of the oilseed rape variety SY-Harnas (Syngenta, Basel, Switzerland), provided by CETAPP (France), were grown both in transparent-wall rhizoboxes (45 cm high, 30 cm wide, 2.5 cm thick, 3.3 L volume) until 2-leaf stage, and in cylindrical PVC pots (50 cm high, 9 cm diameter, 3.1 L volume) until 4-leaf stage. Plants grown in both types of containers were cultured in a greenhouse at the “Lucio Toniolo” experimental farm of the University of Padua (Legnaro, Padua, NE Italy). Rhizoboxes and pots were filled with a sterilized mixture (48 h in an oven at 105°C) of silty-loam soil (pH 8.4) and fine sand (1:1 w/w). A recommended dose of fertilizers, corresponding to 100 kg N, 150 kg P_2_O_5_ and 300 kg K_2_O per hectare was added and carefully mixed with the substrate before rhizobox/pot filling.

Three fungicidal seed treatments, i.e the conventional fungicide Thiram, Sedaxane alone (Sdx) and Sdx in combination with Fludioxonil and Metalaxyl-M (Flu+Met+Sdx) were investigated under conditions in which the soil had been inoculated with *R. solani* (+ Rhizoctonia) or not (− Rhizoctonia), and results were compared with controls left untreated with fungicides (**Table 1**). Seed dressing was performed at Syngenta (CETAPP, France) by injecting the slurry formulation of fungicides into a chamber equipped with an air influx to allow for thorough seed mixing. The action of adhesive co-formulants ensured a complete and homogeneous distribution of the a.i. dose. This resulted in 8 treatments in both rhizobox and pot trials. The trials were arranged following a completely randomized experimental design with 3 replicates. Each replicate (i.e., rhizobox/pot) consisted of 3 subsamples (individual plants) (Figure S1).

**Table 1:** List of seed treatments applied with or without *R. solani* in both rhizobox and pot trials and the amount of added active ingredients (a.i.) per 1 million (1 M) seeds.

Fungicide treatment	a.i. dose	
	(g)	(mmol)
Untreated (absolute control)	–	–
Thiram	19.9 g	82.8
Sedaxane (Sdx)	2.5 g	7.54
Fludioxonil + Metalaxyl-M + Sedaxane (Flu+Met+Sdx)	5.6 g (2.25+0.85+2.50)	9.06+3.05+7.54

Soil inoculation was performed before sowing by using barley seeds infected with *R. solani* strain RS 22 (AG2-1). Four infected barley seeds were applied to each rhizobox and pot as follows: seeds were pestled in a mortar and the resulting flour was mixed with 1 mL of milliQ water. The inoculum solution was equally divided and added to soil at three equally spaced positions, wherein oilseed rape seeds were sown a few days later. In order to avoid artefacts due to organic matter supply, four infected and sterilized (in autoclave at 120°C for 20 min) barley seeds were similarly applied in the treatments not inoculated (− Rhizoctonia) of both rhizoboxes and pots. As such, the treatments constituting non-treated soils contained inactivated pathogens. After soil inoculation, rhizoboxes and pots were kept at a constant temperature of 15°C for 10 days prior sowing to allow the development of the *Rhizoctonia* mycelium.

Three seeds of oilseed rape per rhizobox and pot were sown at 1 cm depth and equally spaced apart, in correspondence with the positions of pathogen inoculation. Plants were kept in the greenhouse at 20°C/15°C (day/night), 12h/12h (light/dark) and 70% air humidity. The whole rhizoboxes were covered with a black film to avoid any interaction of light with root growth through the transparent walls, and the rhizoboxes were placed at a 45° angle to facilitate un-destructive root observations. Oilseed rape plants were grown until 24 Days After Sowing (DAS; 2-leaf stage) in rhizoboxes and 28 DAS in the pot trial (4-leaf stage). Plants were regularly watered in both rhizoboxes and pots throughout the experiment.

### 2.2 Root growth analysis

Root growth parameters were revealed in the rhizobox trial only. Root depth was measured at 2-day intervals, from 6 to 17 DAS, by means of a ruler leaned on the lower transparent wall of the rhizoboxes. The dynamics of root deepening (Y) was fit with the Gompertz equation, as follows:


$$Y\;=\;a\;e^{-e^{\left(b\;-\;c\;x\right)}}$$

where a (asymptote), b (value of x at ½ a), and c (deepening rate) are coefficients and x is time (DAS).

At the end of the rhizobox trial, plants were harvested and their shoots and roots were separated. The roots were gently washed, separated from soil particles and stored in a 15% v/v ethanol solution until further processing. Root length, surface area and diameter were measured by analysis of 1-bit 300-DPI TIFF images of the root systems acquired through a flatbed scanner (Epson Expression 11000XL, Epson, Suwa, Japan) using the WinRhizo software (Regent Instruments, Ville de Québec, QC, Canada).

The dry weight of the roots was later measured in plants grown both in rhizoboxes and pots, after oven-drying for 48 h at 105°C.

### 2.3 Shoot parameters

Due to their greater development in comparison to plants grown in rhizoboxes, the photosynthetic activity was measured at 27 DAS only in plants grown in pots by means of an infrared gas analyzer LI-6800 (Li-COR Inc., Lincoln, Nebraska, USA). PSII Photosynthetic Efficiency (*Fv′/Fm′*), stomatal conductance, electron transport rate (ETR) and CO_2_ Net Assimilation (A) were determined following Murchie and Lawson (2013).

The *Fv′/Fm′* ratio was measured as an index of efficiency in energy harvesting by the oxidized (open) reaction centres of photosystem II (PSII) in the last developed leaf, where *Fv′* and *Fm′* represent variable and maximal fluorescence, respectively. As *Fm′* includes minimal fluorescence (*F_0_′*) of a dark-adapted leaf, *Fv′* is calculated as *Fm′* – *F_0_′*. Dark adaptation was set with the use of a far-red light to excite photosystem I (PSI), thus forcing electrons to drain from PSII. Only a few seconds of far-red light are needed to obtain this effect. The fluorimeter provides a “dark pulse” routine used to determine F_0_ *′*. Five *Fv′/Fm′* records were registered for each leaf of each replicate.

Leaf area was assessed after plant harvest at the end of both rhizobox and pot trials, by means of the LI-3100C Area Meter (Li-COR Inc., Lincoln, Nebraska, USA). Shoot dry biomass was determined after oven-drying for 48 h at 105°C.

### 2.4 Quantitative PCR of *R. solani* in stem base tissues

In order to quantify the presence of *R. solani* in oilseed rape plants, 1cm long portions of the stem base were collected from each plant grown in the pot trial. Three biological replicates (n = 3) per treatment were analyzed, each obtained by mixing 3 subsamples (plants) from every pot. Two technical replicates were performed on each biological replicate. Genomic DNA was extracted using the DNeasy Plant Mini Kit (Qiagen, Milan, Italy), quantified by agarose gel electrophoresis, and then used as template in a qPCR reaction. The qPCR was performed on a Rotor-Gene Q 2plex (Qiagen) using primers specific for *Brassica napus* ENTH gene (Yang et al., 2014), as the internal housekeeping control gene, and for the *R. solani* ARSF4 gene (Dubey et al., 2016). Primers used are reported in **Table S1**. The reaction mixture of 20 µL contained 10 μL of 2X Rotor-Gene SYBR Green PCR MasterMix (Qiagen), 0.5 μM of each specific primer and 1 μL of DNA template. The qPCR was performed by repeating the following cycle 40 times: 95°C for 30 s; 60°C for 30 s; 72°C for 45 s. Reactions were performed in triplicates.

Relative DNA quantification was performed using the Rotor-Gene v. 2.0.3.2 software and the tool REST (Qiagen).

### 2.5 Statistical analysis

Analysis of variance (ANOVA) was performed using CoStat software (CoHort software, Birmingham, UK) with Student Newmal-Keuls test in order to highlight significant differences among means at p ≤ 0.05, and significance of the main effects “inoculum”, “treatment” and “inoculum × treatment” interaction.

Principal component analysis (PCA) and factorial discriminant analysis (Multigroup Discriminant Analysis (MDA) with Wilks’ lambda and Pillai’s trace tests (Podani, 2007) were carried out using MS Excel XLSTAT (Addinsoft, Paris, France) to describe the response of oilseed rape in pots (4-leaf stage) to fungicide seed treatment and *Rhizoctonia* soil inoculum, in terms of shoot and root growth and photosynthetic parameters. Before analysis, multivariate data normality was verified by the Shapiro test using R 3.0.1 software (Ihaka and Gentleman, 1996), and data were standardized by subtracting the mean and dividing by the standard deviation within each variable.

## 3 Results

### 3.1 Rhizobox trial

#### 3.1.1 Dynamics of root deepening

**Figure 1:**
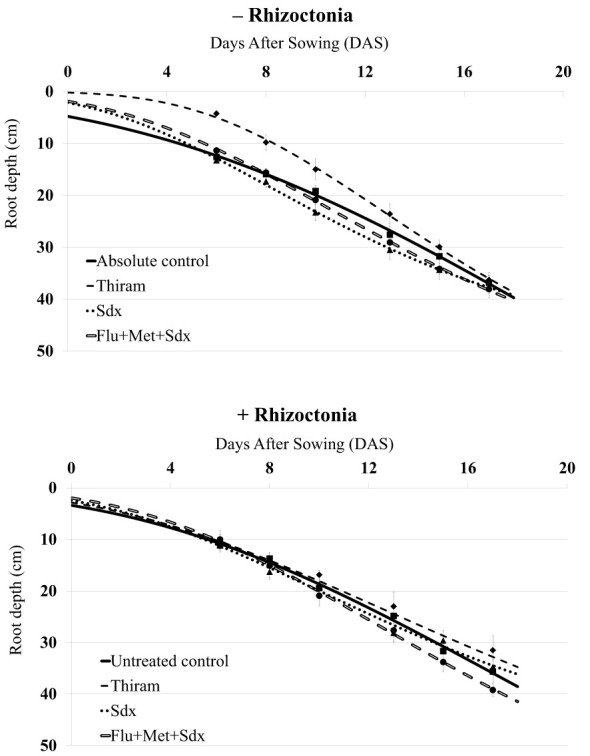
Dynamics of root deepening (cm; mean ± S.E.; n = 3) over 20 days after sowing (DAS) in oilseed rape plants grown in rhizoboxes, in soil inoculated (+ Rhizoctonia) and not inoculated (– Rhizoctonia) with *R. solani*, under three different seed treatments, i.e. Thiram and Sedaxane alone (Sdx), and Sdx in combination with Fludioxonil and Metalaxyl-M (Flu+Met+Sdx). Untreated control comprises plants grown in inoculated soil but without any seed treatment; absolute control comprises plants grown in non-inoculated soil and which received no seed treatment.

The inoculum with *R. solani* did not significantly affect the initial rooting of oilseed rape plants, which reached a maximum root depth of ~40 cm in absence of the pathogen (− Rhizoctonia) and ~35 cm in presence of the pathogen (+ Rhizoctonia) at 17 DAS (**Figure 1**). In non-inoculated soil, after an initial gap, the application of Flu+Met+Sdx and Sdx led to greater root depth as compared to the absolute control starting from 8 DAS. Instead, the use of Thiram caused retarded root deepening within the first 2 weeks (*p* ≤ 0.05), but similar values to controls at the end of the trial.

In *R. solani* inoculated soil (+ Rhizoctonia), only the seed treatment with Flu+Met+Sdx allowed for increased root depth starting from 10 DAS, as compared to all the other treatments. The plants treated with Thiram and Sdx showed substantially similar root deepening pattern to untreated controls (**Figure 1**). According to ANOVA (**Table S2**), the type of treatment had a significant effect (*p* ≤ 0.05), with Sedaxane alone or in combination with the other two a.i. (Flu+Met+Sdx) significantly improving the average root depth in comparison with Thiram over the investigated period.

#### 3.1.2 Root surface area and diameter

**Figure 2:**
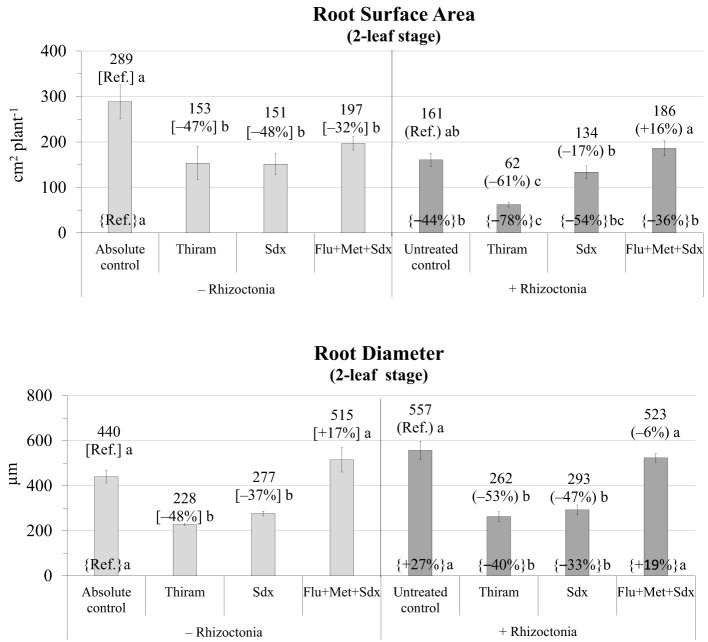
Root surface area (cm^2^ plant^-1^; mean ± S.E.; n=3) and diameter (µm; mean ± S.E; n = 3) of oilseed rape plants at 2-leaf stage (24 days after sowing) grown in rhizoboxes containing soil inoculated (+ Rhizoctonia) and not inoculated (– Rhizoctonia) with *R. solani*, under three different seed treatments, i.e. Thiram, Sedaxane alone (Sdx) and Sedaxane in combination with Fludioxonil and Metalaxyl-M (Flu+Met+Sdx). Different letters indicate significant differences among treatments (Newman–Keuls test, *p* ≤ 0.05) within the same soil conditions, i.e. + Rhizoctonia (round brackets) or – Rhizoctonia (squared brackets), and among + Rhizoctonia treatments and absolute control (brace brackets).

In absence of any fungicide treatment, *R. solani* inoculum significantly reduced the root surface area (−44% vs. absolute control) and increased root diameter (+27%) of oilseed rape plants grown in rhizoboxes (**Figure 2**; **Table S2**).

The two main fixed effects treatment and inoculum were statistically significant (*p* ≤ 0.001) in explaining root surface area, as well as their interaction (*p* ≤ 0.05) (**Table S2**). In particular, in not inoculated soil, root surface area was significantly reduced by all the fungicide treatments, with Thiram and Sedaxane having the most detrimental effect (–47% and –48% respectively, vs. absolute control, *p* ≤ 0.05). Thiram also induced a significant reduction in root surface area in inoculated soil (–61% vs. untreated control, *p* ≤ 0.05), Sedaxane a slight reduction (-17%), and Flu+Met+Sdx a slight increase (+16%) but which were both not statistically different from the untreated control.

Similarly, the ANOVA showed that both Inoculum (*p* ≤ 0.05) and Treatment (*p* ≤ 0.001) also strongly affected root diameter (**Table S2**). Root diameter was markedly (*p* ≤ 0.05) reduced by Thiram and Sdx treatments in both inoculated (–48% and –37%, respectively vs. absolute control) and not inoculated soil (–53% and –47%, respectively vs. untreated control). A full recovery of root diameter was recorded with the use of Sdx in combination with Fludioxonil+Metalaxyl-M (Flu+Met+Sdx), even leading to slight improvements vs. not inoculated soil (+19% vs. absolute control) (**Figure 2**).

#### 3.1.3 Shoot growth parameters

**Figure 3:**
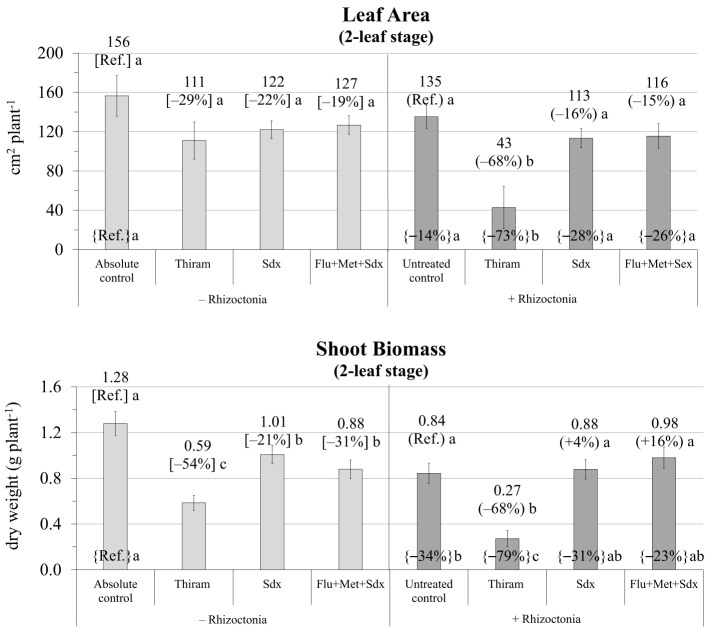
Leaf area (cm^2^ plant^-1^; mean ± S.E.; n=3) and shoot biomass (g dry weight plant^-1^; mean ± S.E.; n = 3) of oilseed rape plants at 2-leaf stage (24 days after sowing) grown in rhizoboxes, containing soil inoculated (+ Rhizoctonia) and not inoculated (– Rhizoctonia) with *R. solani* under three different seed treatments, i.e. with Thiram, Sedaxane alone (Sdx) and Sedaxane in combination with Fludioxonil and Metalaxyl-M (Flu+Met+Sdx). Different letters indicate significant differences among treatments (Newman–Keuls test, *p* ≤ 0.05) within the same soil conditions, i.e. + Rhizoctonia (round brackets) or – Rhizoctonia (squared brackets), and among + Rhizoctonia treatments and absolute control (brace brackets).

The ANOVA revealed that leaf area and shoot biomass were significantly affected by both Treatment (*p* ≤ 0.001) and Inoculum and their interaction (*p* ≤ 0.05) (**Table S2**). Without any seed treatment, inoculum with *R. solani* significantly reduced the leaf area (–14% vs. absolute control) and particularly the shoot dry biomass (–34%) of oilseed rape plants cultivated in rhizoboxes (**Figure 3**; **Table S2**). Leaf area was only slightly reduced (*p* > 0.05) with all the fungicides in both not inoculated and *R. solani* inoculated soil however, while Thiram showed marked phytotoxicity in infected soil (–68% vs. untreated control, *p* ≤ 0.05).

As regards shoot biomass, a similar trend as leaf area was observed; a significant decrease by Thiram in both inoculated (–68% vs. untreated control) and non-inoculated soil (–54% vs. absolute control). Under soil infection, Sedaxane alone and in combination with other fungicides (Flu+Met+Sdx) allowed an appreciable recovery of shoot dry biomass, which was statistically similar to the absolute control (**Figure 3**).

### 3.2 Pot trial

#### 3.2.1 Root biomass

**Figure 4:**
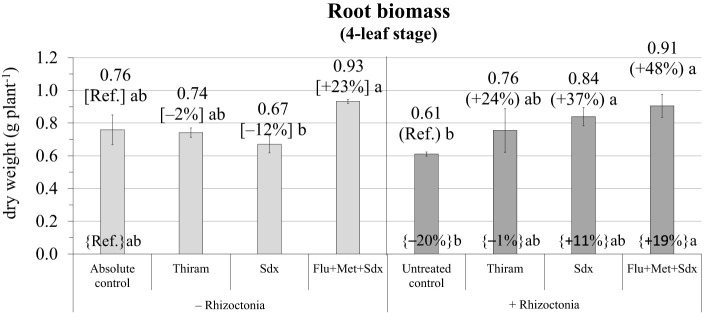
Root biomass (g dry weight plant^-1^; mean ± S.E.; n = 3) of oilseed rape plants at 4-leaf stage (28 days after sowing) grown in pots, containing soil inoculated (+ Rhizoctonia) and not inoculated (– Rhizoctonia) with *R. solani*, under three different seed treatments, i.e. Thiram, Sedaxane alone (Sdx) and Sedaxane in combination with Fludioxonil and Metalaxyl-M (Flu+Met+Sdx). Different letters indicate significant differences among treatments (Newman–Keuls test, *p* ≤ 0.05) within the same soil conditions, i.e. + Rhizoctonia (round brackets) and – Rhizoctonia (squared brackets), and among + Rhizoctonia treatments and absolute control (brace brackets).

According to the ANOVA in the pot trial, the effect of Treatment and the Treatment × Inoculum interaction both had a significant effect on root biomass (*p* ≤ 0.05) (**Table S3**). Indeed, at the later growth stage of 4 leaves in the pot trail, root biomass of plants cultivated in soil infected with *R. solani* and without any fungicide treatment (untreated control) was still reduced (–20% vs. absolute control) (**Figure 3**; **Table S3**). The application of Sedaxane in combination with Fludioxonil and Metalaxyl-M (Flu+Met+Sdx) allowed maximal root dry weight both in *R. solani* inoculated (+48% vs. untreated control, *p* ≤ 0.05) and non-inoculated soil (+19% vs. absolute control). In inoculated soil (+ Rhizoctonia), also the application of Sedaxane alone (Sdx) significantly increased root dry biomass by +37% (*p* ≤ 0.05) (**Figure 4**).

#### 3.2.2 qPCR Quantification of *R. solani* in the Stem Base Tissues

**Figure 5:**
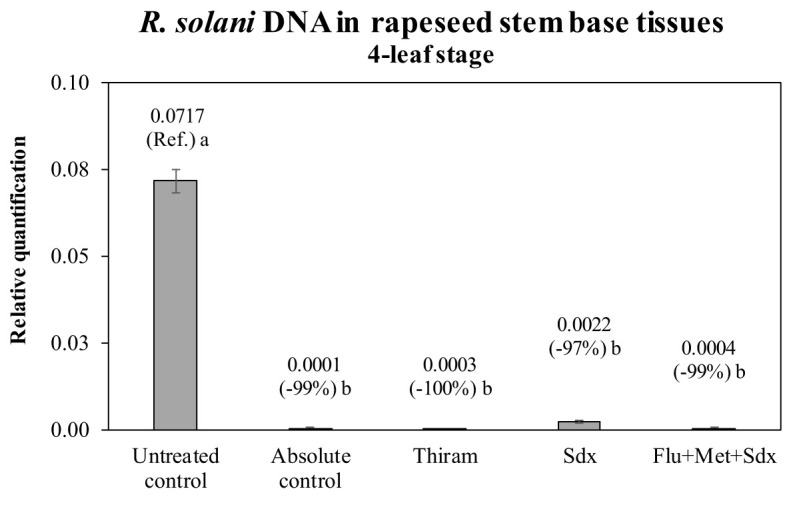
Relative quantification of *R. solani* DNA (mean ± S.E.; n = 3) in stem base tissues of oilseed rape plants at the 4-leaf stage (28 days after sowing) grown in pots containing soil inoculated with *R. solani* (+ Rhizoctonia), under three different seed treatments, i.e. Thiram, Sedaxane alone (Sdx) and Sedaxane in combination with Fludioxonil and Metalaxyl-M (Flu+Met+Sdx). Untreated control comprises plants grown in inoculated soil but without any seed treatment; absolute control comprises plants grown in non-inoculated soil and which received no seed treatment. Different letters indicate significant differences among treatments (Newman–Keuls test, *p* ≤ 0.05).

The presence of *R. solani* DNA was quantified in the stem base tissue of oilseed rape plants of the pot trial. The quantification was carried out in plants grown in inoculated soil (+ Rhizoctonia) and which had received a seed treatment, and compared to untreated plants as well as the absolute control (– Rhizoctonia). All the fungicides drastically reduce the presence of the fungal DNA in the plant tissues (**Figure 5**). There was an absence of visible (shoot) symptoms/injuries by *Rhizoctonia* on all plants regardless of treatment and soil condition. Compared to untreated controls, Thiram and Flu+Met+Sdx reduced the amount of fungal DNA by ~99% leading to a complete absence of *R. solani* DNA, similarly to the absolute control, where the pathogen was never applied to the soil. Sedaxane was also very effective at reducing the presence of *R. solani* DNA by ~97% (**Figure 5**).

#### 3.2.3 Shoot parameters

**Figure 6:**
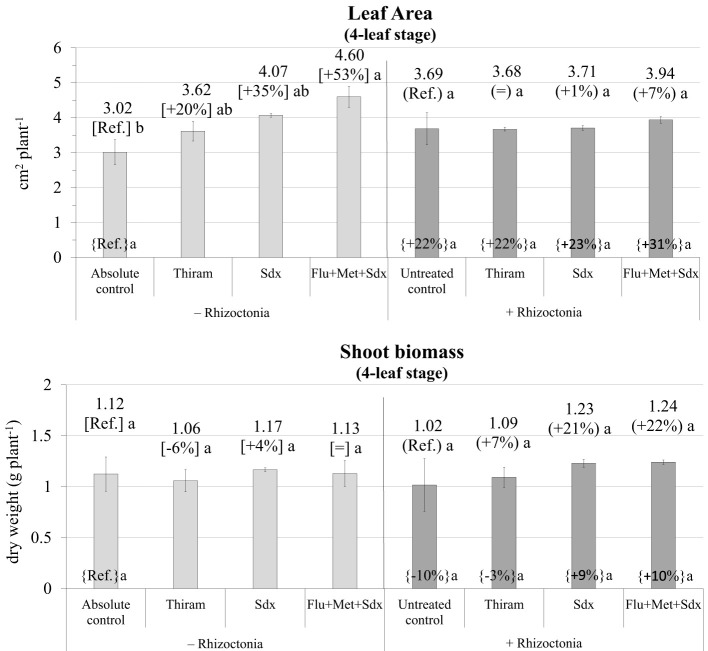
Leaf Area (cm^2^ plant^-1^; mean ± S.E.; n=3) and shoot biomass (g dry weight plant^-1^; mean ± S.E.; n = 3) of oilseed rape plants at 4-leaf stage (28 days after sowing) grown in pots containing soil inoculated (+ Rhizoctonia) and not inoculated (– Rhizoctonia) with *R. solani* under three different seed treatments, i.e. with Thiram, Sedaxane alone (Sdx) and Sedaxane in combination with Fludioxonil and Metalaxyl-M (Flu+Met+Sdx). Different letters indicate significant differences among treatments (Newman–Keuls test, *p* ≤ 0.05) within the same soil conditions, i.e. + Rhizoctonia (round brackets) or – Rhizoctonia (squared brackets), and among + Rhizoctonia treatments and absolute control (brace brackets).

The ANOVA disclosed Treatment as being the only fixed effect with a significant (*p* ≤ 0.05) effect on leaf area (**Table S3**). Indeed, oilseed rape plants grown in non-inoculated soil (– Rhizoctonia) showed increased leaf area at the 4-leaf stage following seed treatment with all the tested fungicides. In particular, significant biostimulation by Sedaxane in combination with Fludioxonil and Metalaxyl-M was observed (+53%; *p* ≤ 0.05) (**Figure 6**). However, no effect of fungicide treatment was disclosed under conditions of soil infection, although treatment with Flu+Met+Sdx slightly, but insignificantly, increased leaf area compared to untreated controls (+7%, *p* > 0.05).

As regards shoot biomass, the ANOVA did not reveal any significant effect from neither Inoculum nor Treatment (**Table S3**). In fact, no significant variations were observed in shoot dry biomass regardless of soil inoculation and fungicide treatment. However, with soil inoculation, both Sdx and Flu+Met+Sdx treatments increased shoot biomass by -20% compared to untreated controls although this was statistically not significant (*p* > 0.05).

#### 3.2.4 Leaf photosynthetic efficiency

**Figure 7:**
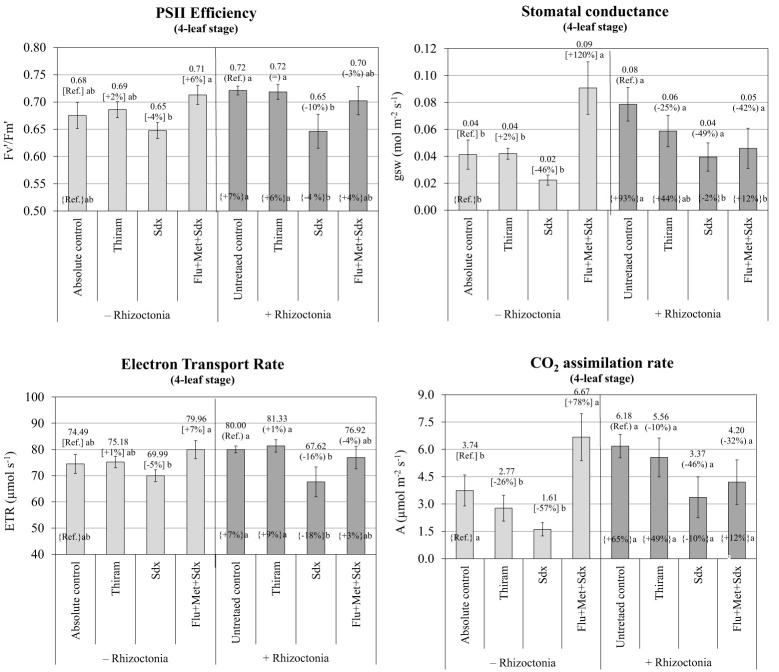
Photosynthetic efficiency of photosystem II (PSII) (*Fv′/Fm′*), stomatal conductance (gsw), electron transport rate (ETR) and CO_2_ assimilation rate **(A)** (mean ± S.E.; n=3) of oilseed rape plants at 4-leaf stage (28 days after sowing) grown in pots containing soil inoculated (+ Rhizoctonia) and not inoculated (– Rhizoctonia) with *R. solani* under three different seed treatments, i.e. with Thiram, Sedaxane alone (Sdx) and Sedaxane in combination with Fludioxonil and Metalaxyl-M (Flu+Met+Sdx). Different letters indicate significant differences among treatments (Newman–Keuls test, *p* ≤ 0.05) within the same soil conditions, i.e. + Rhizoctonia (round brackets) or – Rhizoctonia (squared brackets), and among + Rhizoctonia treatments and absolute control (brace brackets).

According to the ANOVA, PSII efficiency, expressed as ratio between variable and maximum fluorescence (*Fv′/Fm′*), was significantly affected by Treatment (*p* ≤ 0.001) and to a lesser extent by Inoculum (*p* ≤ 0.05) at the 4-leaf stage. Neither Inoculum nor Treatment main effects significantly affected ETR or any other physiological parameter (**Table S3**). The efficiency of photosystem II, and the electron transport rate were fairly stable across seed treatments, with no significant effect from *Rhizoctonia* inoculation. Only Sedaxane applied alone reduced the two parameters within a range from –4% to –5% in non-inoculated (*p* > 0.05; n.s.) and from –10% to –16% in inoculated soil (*p ≤*0.05) for the PSII efficiency and ETR respectively. An increase trend was observed in the PSII efficiency and ETR (*p* > 0.05) when the Flu+Met+Sdx seed treatment was given to plants grown in non-inoculated soil (**Figure 7**).

A similar trend was observed in regards to the variation in stomatal conductance (gsw) and CO_2_ assimilation rate (A) in non-inoculated soil (– Rhizoctonia). Here, a +120% (*p ≤*0.05) and +78% (*p* ≤ 0.05) increase followed seed treatment with Sedaxane in combination with Fludioxonil and Metalaxyl-M for gsw and CO_2_ assimilation rate respectively (**Figure 7**). In this soil condition, the use of Sedaxane alone was instead associated with a relevant decrease, but insignificant, of both stomatal conductance (–46%) and CO_2_ assimilation rate (-57%) in comparison to the absolute control, and lower and not significant variations were observed with Thiram.

Noteworthy, soil inoculation by *R. solani* allowed for a relevant increase in both stomatal conductance (+93%; *p ≤*0.05) and CO_2_ assimilation rate (+65%; *p* > 0.05) as compared to the absolute control. Under soil infection, a decrease of both stomatal conductance and CO_2_ assimilation was revealed following all the fungicide treatments, in particular with Sedaxane alone and in a mixture (Flu+Met+Sdx). However, these treatments merely permitted stomatal conductance and CO_2_ assimilation rates to reach comparable levels to those of the absolute control.

#### 3.2.5 MDA and PCA

**Figure 8:**
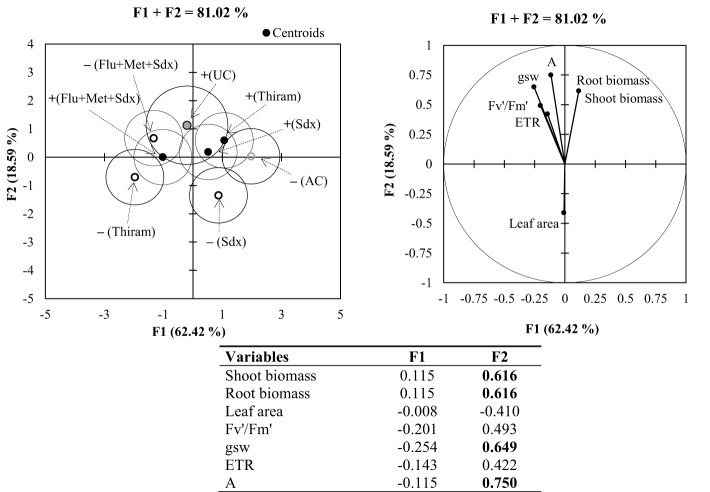
Multigroup discriminant analysis (MDA; left) and principal component analysis (PCA; right) for shoot and root parameters of oilseed rape plants at 4-leaf stage (28 days after sowing) grown in pots, containing soil inoculated (+ in the MDA) and not inoculated (– in the MDA) with *R. solani* under three different seed treatments, i.e. Thiram, Sedaxane alone (Sdx) and Sedaxane in combination with Fludioxonil and Metalaxyl-M (Flu+Met+Sdx). In MDA: UC: untreated control (+*Rhizoctonia*), AC: absolute control (–*Rhizoctonia*). In the PCA: *Fv′/Fm′*: photosynthetic efficiency of photosystem II (PSII); gsw: stomatal conductance; ETR: electron transport rate; A: CO_2_ assimilation rate. The isodensity confidence circles contain 75% of the variability. In the bottom table, highly informative variables (loadings > |0.5|) highlighted in bold, within synthetic variables F1 and F2.

Principal Component Analysis (PCA) applied to the pot trial allowed for the identification of two synthetic variables, F1 (64.42%) and F2 (18.59%), which explained an overall variability of 81.02% (**Figure 8**). The most significant variables (loadings > |0.5|) were shoot and root biomass, stomatal conductance (gsw) and CO_2_ assimilation rate (A) associated to F2. According to the vector direction of each variable in PCA, there is strong positive correlation among all the parameters related to the photosynthetic activity, and a clear negative correlation between shoot dry biomass and leaf area.

According to the centroids position and cluster separation in MDA, a different plant response was observed under the investigated treatments. Within non-inoculated soil, the effects of seed treatment with Sedaxane in combination with Fludioxonil and Metalaxyl-M (Flu+Met+Sdx) was mostly related to variations in physiological parameters of leaf photosynthesis. Instead, the effects of Thiram and Sedaxane alone were mostly associated to variations in plant biomass (both root and shoot) under soil inoculation, and leaf area in non-inoculated soil.

## 4 Discussion

The use of antifungal ingredients for seed treatment has been receiving increasing interest in recent years not only due to their effective control of seed- and soil-borne pathogens during early growth stages, but also due to their possible biostimulant properties, particularly on plant roots. Among recently released fungicides, Sedaxane has proven to have a broad-spectrum activity, particularly in regards to *R. solani* and *Mycosphaerella reliana* (Swart, 2011; Dal Cortivo et al., 2017) growth control, and its registration approval in cereal crops is granted more frequently worldwide.

In the present study, we innovatively investigated the effects of Sedaxane on oilseed rape, as a first example of application in seed treatment in a dicotyledonous species. This was done to compare its effects with already existing and commercially available fungicides. The objective to distinguish the protective effect from possible biostimulant properties is also innovative, with this approach being scarcely applied in literature. Accordingly, the trials were conducted in *R. solani* inoculated and non-inoculated soil of previously sterilized substrate. It cannot be ruled out however, that sterilization may have altered plant growth to a certain extent due to inactivation of soil microbes.

We here demonstrate that all the investigated fungicides efficiently protected against *R. solani*; the level of *Rhizoctonia* DNA at plant collar was reduced by at least 97%. This justifies the absence of visible symptoms of pathological infection on the plants. Since roots are the main plant organ targeted by *Rhizoctonia*, without seed protection the infection caused marked root growth impairments (–45% in surface area at 2 leaves and –20% of biomass at 4 leaves). The full recovery of root biomass at the 4-leaf stage in plants that received seed treatment therefore illustrates the effectiveness of the formulations and doses of all tested fungicides.

The possession of secondary biostimulation effects in the fungicides were assessed as additional growth compared with the absolute control (no *Rhizoctonia*, no seed treatment). As such, Sedaxane mixed with Fludioxonil and Metalaxyl-M had ultimately (4-leaf stage, end of the trial) a clear root biostimulating power, as conveyed by the increased root biomass in both the *R. solani* inoculated soil and non-inoculated soil. This might be due to a synergistic effect of the three fungicides due to their differing modes of action (MOA) and target sites in pathogens. Indeed, Fludioxonil is a preventive fungicide, while Metalaxyl-M and Sedaxane have systemic activity. Fludioxonil targets the histidine kinase enzyme involved in osmotic signal transduction (MOA E2), Metalaxyl-M the RNA polymerase I enzyme (MOA A1), while Sedaxane is a succinate dehydrogenase inhibitor (SDHI) blocking fungus respiration (MOA C2). When applied alone, Sedaxane showed significant biostimulating effects on root biomass under *Rhizoctonia*-inoculated soil, but not in non-inoculated soil. Indeed, sedaxane has already been reported to have auxin- and gibberellin-like activities in various cereals crops (Zeun et al., 2013; Dal Cortivo et al., 2017). Auxins are known to exert a central role in primary root elongation, lateral root initiation, and root hair development, and this is expected to be the main mechanism of root stimulation by Sedaxane. The gibberellic activity, on the other hand, could be responsible for better shoot growth and leaf expansion (Fleet and Sun, 2005), which are both strategic in the open field to ensure fast plant establishment in the sensitive early phases of growth. Although in early oilseed rape stages (2 leaves) all the tested fungicide formulations exerted a significant shoot and root phytotoxicity, within the 4-leaf stage a full growth recovery was ascertained. At this time, Sedaxane either alone and in a mixture with other fungicides allowed improved shoot growth of oilseed rape particularly under *Rhizoctonia* pressure, possibly due to its gibberellinic-like activity. In the same way, in non-inoculated soil, improved leaf expansion, PSII efficiency and stomatal conductance could be observed as the main beneficial effects of the Sedaxane fungicide mixture.

The initial shoot and root phytotoxicity was associated particularly to the use of Thiram, possibly due to its higher dosage as compared to the other a.i. used in these trials. An over-dosage of fungicides has been reported to negatively affect plant growth and development, with evident alteration of plant physiological processes, such as nitrogen metabolism and photosynthetic activity (Saladin et al., 2003; Petit et al., 2008). For instance, the application of strobilurin analogs was found to reduce the *Fv′/Fm′* fluorescence ratio in soybean, linked to an electron transport block from PSII to PSI (Nason et al., 2007). A decrease in net photosynthesis was also correlated to an increase in fungicide doses in grapevine and pea (Saladin et al., 2003; Nason et al., 2007). Indeed, fungicides could reduce the activity of enzymes involved in the synthesis of chlorophyll and other foliar pigments (i.e., carotenoids), thus causing reduced CO_2_ assimilation, and thereby biomass accumulation and yield (Shahid et al., 2018). These effects were clearly found at 2-leaf stage in our rhizobox trial, with reduced shoot biomass as well as leaf area with the use of Thiram, regardless of soil inoculation.

Although the initial phytotoxicity was found to be transiently affecting oilseed rape with the use of all the tested fungicide formulations, the reasons as to why root growth was retarded (but to a lesser extent shoot) with Sedaxane use in either lone use or in mixture, in absence of soil inoculation remain unclear. In previous studies (Rose et al., 2018; Shahid et al., 2018), decreases in shoot biomass in pea and lupin were associated with a modification or inhibition of the activity of various enzymes involved in growth, development and metabolism when treated with systemic fungicides, such as Kitazin, Hexaconazole and Carbendazim, following their translocation from root to shoot through the xylematic sap flow. This cannot be the case for Thiram as it is not a systemic a.i. Indeed it is thought that fungicide phytoxicity is related to cellular damage and production of Reactive Oxygen Species (ROS), which can reduce synthesis of lipids, proteins, and nucleic acids, and provoke inefficient water and nutrient uptake (Yilmaz et al., 2017; Shahid et al., 2018). This would explain transient below-ground phytotoxicity of Sedaxane in correspondence with the penetration/uptake sites of the fungicide, even in absence of pathogen pressure. We therefore suspect that oilseed rape is highly sensitive to seed treatments with fungicides, regardless of a.i. choice, and the observed growth retard was likely exacerbated by the small soil volume in rhyzoboxes used for assessing plant growth at the early stage.

Despite efficient exclusion of *Rhizoctonia* DNA from the stem base tissues of oilseed rape, the presence of mycelium in the rhizosphere is also hypothesized to secrete toxic compounds which affect root growth and development in the host plant. However, this requires further specific investigations to be confirmed. Through secondary metabolism, *R. solani* can produce several compounds that aid the infection process, such as fatty acids, steroids, phenolic compounds and glycoproteins, all of which are also associated to root phytotoxicity (Mandava et al., 1980; Adachi and Inagaki, 1988; Velazhahan and Vidhyasekaran, 2000; Ma et al., 2004; Aliferis and Jabaji, 2010). However, Sedaxane in combination with Fludioxonil and Metalaxyl-M, was capable of partially recovering the root surface area at the 2-leaf stage under *R. solani* pressure.

The question which arises is why root biostimulation with Sedaxane is better expressed under *Rhizoctonia* soil infection rather than without the pathogen. Actually, we argue that in presence of this pathogen a priming effect probably exists in oilseed rape plants, which involves a physiochemical response capable of corroborating the biostimulant effect of the fungicide. Priming is known to activate systemic defense responses only when the plant is challenged by a pathogen or is exposed to abiotic stress (Aranega-Bou et al., 2014). Since natural or synthetic chemicals and infection by pathogens can induce priming effects in plants (Aguado et al., 2019; Westman et al., 2019; De Vega et al., 2021), we hypothesize that the greater positive effect observed with the use of Sedaxane alone, and to a greater extent when in combination with Fludioxonil and Metalaxil-M and in the presence of *R. solani* could be ascribed to a plant response related to priming. While this hypothesis requires further investigation to clarify the possible involved mechanisms, Sedaxane in combination with Fludioxonil and Metalaxyl-M can be agronomically exploited in a dicot species such as oilseed rape to control *Rhizoctonia*. A faster growth within the 4-leaf stage would also allow oilseed rape to quickly overcome the initial delicate phases of growth, with possible improved tolerance against abiotic stresses as well. Soil sterilization of rhizoboxes and pot trials was necessary to disentangle the fungal protection properties from the biostimulating effects of Sedaxane, while further investigations in real field conditions will be necessary to ascertain possible interactions with the soil microbiota and the molecular cross-talk of biopriming.

## 5 Conclusions

Seed treatment with Sedaxane is consolidating in cereal crops, showing successful control of a wide spectrum of fungal pathogens, particularly *R. solani*, which can be exploited agronomically. Here we confirm an efficient control of this pathogen also in the dicot oilseed rape by Sedaxane either alone or in combination with Fludioxonil and Metalyl-M. Commercial formulations with low contents of these a.i are good candidates for replacing the over-used conventional fungicide Thiram, which is being withdrawn in various countries worldwide. Although all the fungicides tested here had initial phytotoxicity, Sedaxane exhibited clear root biostimulant properties as a supplementary effect to its antifungal features, like similarly observed in cereal crops. This suggests new opportunities for exploiting the biostimulant value of this fungicide in other *Brassicaceae* species, such as turnip, cabbage, kale, and leaf rape. The optimal biostimulant effects of Sedaxane are observed in combination with other a.i. and under high *Rhizoctonia* pressure however. As seed treatments require small amounts of fungicides in the field, the discovery of new a.i. or natural compounds with double effects at low doses is expected to reduce the impact of agrochemicals on the environment. This is advantageous both for the agricultural industry and for improving sustainable practices.

Our preliminary results suggest that young oilseed rape seedlings are highly sensitive to seed treatments with fungicides, particularly Thiram, but the growth retardation is quickly overcome within the 4-leaf stage. At this developmental stage, the biostimulant properties could be observed aboveground as well. While fungicide biostimulation is associated with improved photosynthetic efficiency and stomatal conductance in absence of *Rhizoctonia*, further investigations will be necessary to clarify the physiological mechanisms sustaining the maximal stimulating effect under high pathogen pressure and whether these results are confirmed in the open field.

## Data availability statement

The raw data supporting the conclusions of this article will be made available by the authors, without undue reservation.

## Author contributions

Conceptualization, TV: Methodology, TV and LS: Investigation, FC, ADS: Resources, TV: Data curation, FC, ADS. and GB: Writing—original draft preparation, FC, ADS and AP: Writing-review and editing, AP, SSM, LS and TV: Supervision, TV: Project administration, TV: Funding acquisition, TV. All authors contributed to the article and approved the submitted version.
