# Supplementary material for: Early morpho-physiological response of oilseed rape under seed applied Sedaxane fungicide and Rhizoctonia solani pressure
Source: Front Plant Sci. 2023 Feb 22;14:1130825. doi: 10.3389/fpls.2023.1130825 (PMC9992717; doi:10.3389/fpls.2023.1130825)
Supplement: Supplementary file 1 [file DataSheet_1.docx]

Supplementary Material

Early morpho-physiological response of oilseed rape under seed applied Sedaxane fungicide and *Rhizoctonia solani* pressure

Anna Panozzo*, Giuseppe Barion, Selina Sterup Moore, Francesca Cobalchin, Alberto Di Stefano, Luca Sella, Teofilo Vamerali

*** Correspondence:**Anna Panozzo
[anna.panozzo@unipd.it](mailto:anna.panozzo@unipd.it)

# Supplementary Figures and Tables

**Figure S1.** (a) Rhizoboxes at 14 days after sowing (DAS) with maximum root depth (dotted line) of oilseed rape in soil inoculated (+ Rhizoctonia) and not inoculated (– Rhizoctonia) with *R. solani*, under three different seed treatments, i.e. Thiram, Sedaxane alone (Sdx), and Sedaxane in combination with Fludioxonil and Metalaxyl-M (Flu+Met+Sdx); (b) an overview of the pot trial at 7 DAS.

**Table S1**. Primers used for qPCR quantification of *R. solani* in plant stem base tissues of oilseed rape.

| Primer name | Primer sequence | Product size |
| --- | --- | --- |
| ENTH_f: | GTTTAGACCCGTTGCTGCTC | 250 bp |
| ENTH_/r: | TTGTCCATCTCAGCCATTTG |  |
| ARSF4_f: | CAACGGATCTCTTGGCTCTC | 317 bp |
| ARSR4_r: | GGTGTCCTCGGCGATAGATA |  |

**Table S2.** Analysis of variance (ANOVA) for root and shoot parameters of oilseed rape plants at 2-leaf stage in rhizoboxes containing soil inoculated (+ Rhizoctonia) and not inoculated (– Rhizoctonia) with *R. solani*, under three different seed treatments, i.e. Thiram, Sedaxane alone (Sdx), and Sedaxane in combination with Fludioxonil and Metalaxyl-M (Flu+Met+Sdx). Different letters indicate significant differences among treatments within the same parameter according to the Student Newman-Keuls test (*p* ≤ 0.05).

| **Main factors** |  | **Root parameters** | | | | | | | |  | | **Shoot parameters** | | | | |
| --- | --- | --- | --- | --- | --- | --- | --- | --- | --- | --- | --- | --- | --- | --- | --- | --- |
|  |  | **Depth** | |  | **Surface area** | |  | **Diameter** | |  | **Leaf area** | | |  | **Shoot Biomass (DW)** | |
|  |  | (cm) | |  | (cm^2^ plant^-1^) | |  | (µm) | |  | (cm^2^ plant^-1^) | | |  | (g plant^-1^) | |
| **Inoculum** |  |  |  |  |  |  |  |  |  |  |  | |  |  |  |  |
|  |  |  |  |  |  |  |  |  |  |  |  | |  |  |  |  |
| - *Rhizoctonia* |  | 24.9 | a (ref.) |  | 195 | a (ref.) |  | 346 | b (ref.) |  | 128 | | a (ref.) |  | 0.92 | a (ref.) |
| + *Rhizoctonia* |  | 24.5 | a (-2%) |  | 137 | b (-30%) |  | 392 | a (+13%) |  | 103 | | b (-20%) |  | 0.79 | b (-14%) |
|  |  |  |  |  |  |  |  |  |  |  |  | |  |  |  |  |
| **Treatment** |  |  |  |  |  |  |  |  |  |  |  | |  |  |  |  |
|  |  |  |  |  |  |  |  |  |  |  |  | |  |  |  |  |
| Control |  | 24.5 | Ab (ref.) |  | 225 | A (ref.) |  | 505 | A (ref.) |  | 146 | | A (ref.) |  | 1.01 | A (ref.) |
| Thiram |  | 22.1 | b (-10%) |  | 96 | c (-51%) |  | 248 | b (-51%) |  | 77 | | b (-47%) |  | 0.44 | b (-56%) |
| Sdx |  | 25.7 | a (+5%) |  | 144 | b (-44%) |  | 285 | b (-44%) |  | 118 | | a (-19%) |  | 0.95 | a (-6%) |
| Flu+Met+Sdx |  | 25.5 | a (+4%) |  | 192 | a (-1%) |  | 502 | a (-1%) |  | 121 | | a (-17%) |  | 0.93 | a (-8%) |
|  |  |  |  |  |  |  |  |  |  |  |  | |  |  |  |  |
| **Significance** |  |  |  |  |  |  |  |  |  |  |  | |  |  |  |  |
|  |  |  |  |  |  |  |  |  |  |  |  | |  |  |  |  |
| Inoculum (I) |  |  | ns |  |  | *** |  |  | * |  |  | | ** |  |  | * |
| Treatment (T) |  |  | * |  |  | *** |  |  | *** |  |  | | *** |  |  | *** |
| I × T |  |  | ns |  |  | * |  |  | ns |  |  | | ns |  |  | * |
|  |  |  |  |  |  |  |  |  |  |  |  | |  |  |  |  |

ns: not significant; *: significant at *p* ≤ 0.05; **: significant at *p* ≤ 0.01; ***: significant at *p* ≤ 0.001

**Table S3.** Analysis of variance (ANOVA) for root and shoot parameters of oilseed rape plants at 4-leaf stage in pots containing soil inoculated (+ Rhizoctonia) and not inoculated (– Rhizoctonia) with *R. solani*, under three different seed treatments, i.e. Thiram, Sedaxane alone (Sdx), and Sedaxane in combination with Fludioxonil and Metalaxyl-M (Flu+Met+Sdx). Different letters indicate significant differences among treatments within the same parameter according to the Student Newman-Keuls test (*p* ≤ 0.05).

| **Treatment** |  | **Roots** | |  | **Shoots** | | | | | | | | | | | | | | | | |
| --- | --- | --- | --- | --- | --- | --- | --- | --- | --- | --- | --- | --- | --- | --- | --- | --- | --- | --- | --- | --- | --- |
|  |  | **Biomass (DW)** | |  | **Leaf area** | |  | **Biomass (DW)** | |  | **PSII Efficiency** | |  | **Stomatal conductance** | |  | **ETR** | |  | **A** | |
|  |  | (g plant^-1^) | |  | (cm^2^ plant^-1^) | |  | (g plant^-1^) | |  | (Fv'/Fm') | |  | (mol m^-2^ s^-1^) | |  | (µmol s^-1^) | |  | (µmol m^-2^ s^-1^) | |
| **Inoculum** |  |  | |  |  | |  |  | |  |  | |  |  | |  |  | |  |  | |
|  |  |  | |  |  | |  |  | |  |  | |  |  | |  |  | |  |  | |
| *- Rhizoctonia* |  | 0.78 | a (ref.) |  | 3.82 | a (ref.) |  | 1.12 | a (ref.) |  | 0.68 | b (ref.) |  | 0.05 | a (ref.) |  | 74.91 | a (ref.) |  | 3.70 | a (ref.) |
| *+ Rhizoctonia* |  | 0.75 | a (-4%) |  | 3.75 | a (-2%) |  | 1.15 | a (+3%) |  | 0.71 | a (+4%) |  | 0.05 | a (=) |  | 76.23 | a (+2%) |  | 4.73 | a (+28%) |
|  |  |  | |  |  | |  |  | |  |  | |  |  | |  |  | |  |  | |
| **Treatment** |  |  | |  |  | |  |  | |  |  | |  |  | |  |  | |  |  | |
|  |  |  | |  |  | |  |  | |  |  | |  |  | |  |  | |  |  | |
| Control |  | 0.64 | b (ref.) |  | 3.35 | b (ref.) |  | 1.07 | a (ref.) |  | 0.70 | a (ref.) |  | 0.06 | a (ref.) |  | 76.85 | a (ref.) |  | 4.78 | a (ref.) |
| Thiram |  | 0.75 | b (+17%) |  | 3.65 | ab (+9%) |  | 1.08 | a (+1%) |  | 0.71 | a (+1%) |  | 0.05 | a (-17%) |  | 78.26 | a (+2%) |  | 4.16 | a (-13%) |
| Sdx |  | 0.75 | b (+17%) |  | 3.88 | ab (+16%) |  | 1.20 | a (+12%) |  | 0.64 | b (-9%) |  | 0.03 | a (-50%) |  | 68.80 | a (-10%) |  | 2.49 | a (-48%) |
| Flu+Met+Sdx |  | 0.92 | a (+44%) |  | 4.27 | a (+27%) |  | 1.19 | a (+11%) |  | 0.74 | a (+6%) |  | 0.07 | a (+17%) |  | 78.44 | a (+2%) |  | 5.43 | a (+14%) |
|  |  |  | |  |  | |  |  | |  |  | |  |  | |  |  | |  |  | |
| **Significance** |  |  | |  |  | |  |  | |  |  | |  |  | |  |  | |  |  | |
|  |  |  | |  |  | |  |  |  |  |  | |  |  | |  |  | |  |  | |
| Inoculum (I) |  | ns | |  | ns | |  | ns | |  | * | |  | ns | |  | ns | |  | ns | |
| Treatment (T) |  | ** | |  | * | |  | ns | |  | *** | |  | ns | |  | ns | |  | ns | |
| I × T |  | * | |  | ns | |  | ns | |  | ns | |  | ns | |  | ns | |  | ns | |
|  |  |  | |  |  | |  |  | |  |  | |  |  | |  |  | |  |  | |

ns: not significant; *: significant at *p* ≤ 0.05; **: significant at *p* ≤ 0.01; ***: significant at *p* ≤ 0.001
